# Validity and measurement invariance across sex, age, and education level of the French short versions of the European Health Literacy Survey Questionnaire

**DOI:** 10.1371/journal.pone.0208091

**Published:** 2018-12-06

**Authors:** Alexandra Rouquette, Théotime Nadot, Pierre Labitrie, Stephan Van den Broucke, Julien Mancini, Laurent Rigal, Virginie Ringa

**Affiliations:** 1 Université Paris-Saclay, Univ. Paris-Sud, UVSQ, CESP, INSERM, Villejuif, France; 2 AP-HP, Bicêtre Hôpitaux Universitaires Paris Sud, Public Health and Epidemiology Department, Le Kremlin-Bicêtre, France; 3 Université Paris-Saclay, Univ. Paris-Sud, General Practice Department, Le Kremlin-Bicêtre, France; 4 Université Catholique de Louvain, Louvain, Belgium; 5 Aix-Marseille Univ, INSERM, IRD, UMR1252, SESSTIM, “*Cancers*, *Biomedicine & Society*” group, Marseille, France; 6 APHM, Timone Hospital, Public Health Department (BIOSTIC), Marseille, France; 7 Institut National d’Etudes Démographiques (INED), Paris, France; University of Michigan, UNITED STATES

## Abstract

**Background:**

Short versions of the European Health Literacy Survey (HLS-EU) questionnaire are increasingly used to measure and compare health literacy (HL) in populations worldwide. As no validated versions of these questionnaires have thus far appeared in French, this study aimed to study the psychometric properties of the French translation of the 16- and 6-item short versions (HLS-EU-Q16 and HLS-EU-Q6), including their measurement invariance across sex, age, and education level.

**Methods:**

A consensual French version of the HLS-EU-Q16 and HLS-EU-Q6 was developed by following the current recommendations for transcultural questionnaire adaptation. It was then completed by 317 patients recruited in waiting rooms of general practitioners in the Paris area (France). Structural validity was studied with the Rasch model for the HLS-EU-Q16 and confirmatory factorial analysis (CFA) for the HLS-EU-Q6. Concurrent and convergent validity, respectively, were assessed by scores on the Functional Communicative Critical Health Literacy (FCCHL) questionnaire and the physicians’ evaluations of their patient’s HL.

**Results:**

The 16 items of the HLS-EU-Q16 were Rasch homogenous but meaningful differential item functioning (DIF) was found across sex, age, and/or education level for eight items. The CFA model fit for the HLS-EU-Q6 was poor. The overall scores for both HLS-EU short versions correlated poorly with the FCCHL scores. Similarly, HL levels defined using either short-version score did not agree with physicians’ HL assessments.

**Conclusion:**

The French version of the HLS-EU-Q16 has acceptable psychometric properties, despite meaningful DIF for age, sex and education level and a poor discriminative power among subjects with average to high HL level. We recommend its use to measure HL in populations with sufficient reading skills to discriminate between subjects with low to average HL. Also, sensitivity analyses should be performed to evaluate the potential measurement bias due to DIF. Our results did not demonstrate the validity of the HLS-EU-Q6.

## Introduction

Health literacy (HL) is defined as "the cognitive and social skills which determine the motivation and ability of individuals to gain access to, understand and use information in ways which promote and maintain good health" [1]. Three dimensions are distinguished: functional literacy, which involves basic skills (reading, writing, etc.) to access health information; interactive literacy, which refers to more advanced cognitive skills to understand this information; and critical literacy, which involves in-depth cognitive and social skills that ultimately lead to better control of life events [2]. Low HL has been shown to be associated with poor health, limited survival, and a higher cost of care [3–7]. Furthermore, the World Health Organization emphasizes the central role of HL in addressing health inequalities worldwide [1]. Nevertheless, HL has been studied only sparsely in France, likely due to the lack of adequate measurement instruments validated in French [8–10].

Some screening tools for low HL, such as the Rapid Estimate of Adult Literacy in Medicine (REALM) or the Test of Functional Health Literacy in Adults (TOFHLA) have been translated in French, but they assess only functional literacy, through timed tests evaluating the recognition of medical terms or the understanding of medical texts [11,12]. More recently, however, French adaptations of broader tools are being developed. For instance, the Functional Communicative Critical Health Literacy (FCCHL) scale, based on Nutbeam’s definition, has recently been validated in French, but its use in epidemiological studies is limited because the implication of disease diagnosis in its wording (“If you are diagnosed…”) reduces its relevance in healthy populations [13]. A transcultural adaptation in French of the Health Literacy Questionnaire (HLQ), measuring nine dimensions related to individual traits and abilities as well as contextual and health system resources, has recently been published [14,15]. Another interesting tool is the European Health Literacy Survey Questionnaire (HLS-EU-Q), which is built on a conceptual model of HL developed by a European consortium (not including France) based on a review of 170 publications [16]. This model integrates four health information processing skills (accessing, understanding, appraising, and applying health information) applied in three health contexts (healthcare, disease prevention, and health promotion). These skills go well beyond functional literacy, which focuses mainly on understanding health information; they also consider its communicative (e.g., accessing and discussing this information) and critical dimensions (appraising and applying it) [17,18]. A Delphi method was used to generate and select 47 items covering the 12 domains (three health contexts x four skills) [17].

One of the main obstacles to the use of these questionnaires in epidemiological studies, however, is their length. The addition of more than 40 questions to measure HL is rarely possible in studies that already involve several other questionnaires. Although no short version of the HLQ currently exists, to our knowledge, short versions of the HLS-EU-Q, containing 16 (HLS-EU-Q16) and 6 (HLS-EU-Q6) items [18], have been developed. The 16 items of the HLS-EU-Q16 were selected among the 47 HLS-EU-Q items based on their psychometric properties evaluated by Rasch analysis and their simultaneous good face and content validity by ensuring representation of the 12 HLS-EU domains [19] (S1 Table). This questionnaire provides an overall score of HL that has been shown to be highly correlated (r = 0.82) with the overall score on the full 47-item version of the HLS-EU-Q. The 6 items of the HLS-EU-Q6 were selected from the HLS-EU-Q16 using confirmatory factorial analysis (CFA) to establish its factorial structure, and correlation coefficients with the scores on the longer versions to determine its convergent validity [20]. The HLS-EU-Q6 has been used in a limited number of clinical studies in Europe [21,22], while the HLS-EU-Q16 is increasingly used for population studies in Europe in numerous countries. It has been translated into Dutch [7,23–25], Swedish [26,27], German [28–30], Norwegian [31], Spanish and Catalan [32], Italian [33], Greek [34], Czech [35], Hebrew [36], and Arabic [37]. A French version of this questionnaire has been used in two studies in Belgium [7,38], but the published information regarding its psychometric properties is limited.

French is the fourth most widely spoken first language in the European Union, after German, Italian, and English. It is an official language of four European countries (France, Belgium, Switzerland, and Luxembourg) and of 25 independent nations outside Europe. As the short versions of the HLS-EU-Q are increasingly used to measure and compare HL in populations within Europe and worldwide, it is imperative to ascertain the validity of the French version of these questionnaires. As for any measurement device, measurement invariance is a required property to guarantee accurate group comparisons and is thus essential for questionnaire validation. According to Mokkink et al. and Milsap, “[a] measuring device should function in the same way across varied conditions, so long as those varied conditions are irrelevant to the attribute being measured” [39,40]. The objective of this study was thus to translate the HLS-EU-Q16 and HLS-EU-Q6 into French and to evaluate their psychometric properties, including measurement invariance across sex, age, and education level.

## Methods

### Translation

In accordance with the steps described in the current recommendations on transcultural adaptation of questionnaires [41,42], six experts from various disciplines (epidemiology, biostatistics, psychometrics, general medicine, public health, and psychiatry), including one bilingual English-French expert and five French experts with very high levels of English language proficiency, independently translated the English version of the HLS-EU-Q16 into French [43]. A consensus meeting was then held to arrive at a consensual French version of the questionnaire, based on the six independent translations and on the French version of the questionnaire previously used in Belgium (S1 Text). No back-translation was performed, as this has recently been proven unnecessary [44]. Ten subjects (4 males, mean age = 30 years) tested this version (completion time: 5 to 12 min). No formal cognitive debriefing interviews were performed but short individual discussions to assess acceptability and comprehensiveness of each item. No modification of the translated HSL-EU-Q-16 version was needed after this pilot test. The readability level of the translation, assessed using the Flesch Readability Score adapted to texts written in French, was 48, which corresponds to an undergraduate (bordering end of high-school) level [45].

### Psychometric properties

#### Sample

Subjects were recruited from May 15 to June 30, 2016, in the waiting rooms of 17 general practitioners involved in the general practice network of *Université Paris-Sud* (France). General practitioners were selected to ensure representation of the various social backgrounds that exist in the Paris area, but not statistical representativeness for either Paris or France as a whole. Explanations about the study were provided to all French-speaking patients arriving in the waiting room, aged 18 years or older. They were then asked to complete the “patient questionnaire”. At the end of the day, the physician completed a "physician questionnaire" for each patient who had participated in the study that day. All patients provided signed informed consent to participate. The institutional ethic committee (*Comité d’Ethique du Collège National des Généralistes Enseignants*, n°IRB IRB00010804) approved the study, that is, determined that it met the requirements of legal codes that govern health research in France.

#### Data collection

Patients provided socio-demographic information including sex, age, and education level, as well as perceived health status ("Would you say that overall, your health is: excellent/very good/good/medium/poor?") and perceived financial situation ("Currently, with regard to your household financial situation, would you say that: you are very comfortable/relatively comfortable/just about managing/not really managing <often struggle to make ends meet> or not managing <often have to do without essentials or go deeper into debt>). Patients completed the French version of the HLS-EU-Q16 by indicating their response to each question on a 4-point Likert-like scale ("very easy", "easy", "difficult", "very difficult") for each item. To study the concurrent validity, we measured functional, communicative, and critical HL with the French version of the FCCHL. In addition, the physician answered one question about each patient: “In your opinion, this patient’s level of HL is: inadequate/medium/satisfactory?”. Apart from the World Health Organization’s definition of HL [1], no specific criteria were provided to practitioners to answer this question.

#### Statistical analyses

Answers for each of the 16 items of the HLS-EU-Q16 were re-scored in reverse so that higher scores reflected higher levels of HL. Ceiling and floor effects were identified for each item; these two effects were defined *a priori* as respectively more than 95% of respondents who select the highest and the lowest category.

The structural validity of the 16- and 6-item versions of the HLS-EU-Q was evaluated by using the same statistical strategy used in the initial validation studies [18,20]. Specifically, a Rasch analysis was used for the HLS-EU-Q16, with dichotomized items (the "very easy" category was merged with the "easy" category, and the "difficult" category with the "very difficult" category). A monotonely homogeneous model of Mokken was fitted to verify the three fundamental assumptions (unidimensionality, local independence, monotonicity) on which the Rasch model relies. Its fit was considered acceptable if the Loevinger H coefficients were >0.3 for the H coefficient of scalability and for the H_j_ coefficients associated with each item j (j = 1,…,16) and were >0 for the H_jk_ coefficients associated with each pair of items j and k [46]. The global fit of the Rasch model was evaluated with a Chi^2^ test, and individual item fit with standardized residuals (expected to be ± 2.5) and Chi^2^ tests. The dimensional structure of the HLS-EU-Q6 was studied by using CFA on the reversed 4-point-Likert-like items and as the robust estimator for categorical data being the Weighted least square Means and Variances adjusted. Two models were fitted: a one-factor model and a two-order model with three factors according to health contexts and a higher order factor for global HL. Fit indices used were the comparative fit and Tucker-Lewis indices (CFI & TLI, good fit if >0.95, poor fit if <0.90, acceptable fit elsewhere) and the root mean square error approximation (RMSEA, good fit if <0.06, poor fit if >0.1, acceptable fit elsewhere) [47].

The Rasch analyses allowed us to assess measurement invariance which holds if two subjects being identical on the measured construct but from different groups (males and females, for example) have the same probability of giving any particular answer to any item of the scale [40,48]. If measurement invariance does not hold it means that one or several items of the scale “functions” differently in the groups to be compared (resulting in the Rasch model in different item parameter, termed difficulty, in the two groups) and that group comparisons of the total scale score may be inaccurate; this phenomenon is termed differential item functioning (DIF) [48,49]. Two kinds of DIF can be distinguished: uniform if the relation between the group and the response to the item is identical at every level of the latent trait (HL); otherwise DIF is non-uniform [48]. These both kinds of DIF were investigated in the HLS-EU-Q16 across sex, age (categorized based on tertiles) and educational level (primary or none, secondary, post-secondary) [50]. When statistically significant DIF was observed, the item was split in pseudo-items to estimate its difficulty in each group. DIF was considered meaningful if the difference in item difficulties across groups was higher than 0.25 logit or if more than 25% of the items of the scale were affected by DIF in the same direction. When DIF affected several items in opposite directions, the expected difference in the scale score across groups due to DIF was evaluated [51]. Internal consistency was assessed with the Cronbach alpha coefficient (acceptable if higher than 0.7) [52].

To assess concurrent validity, the overall HLS-EU-Q16 score was computed as the simple sum score of the 16 binary items, while the overall HLS-EU-Q6 score was computed by averaging the responses to the six items on the reversed four-point Likert scale, both as recommended for other language versions [20]. The three levels of HL were the same as those in the other language versions: inadequate (HLS-EU-Q16 score ≤8, HLS-EU-Q6 score ≤2), problematic (HLS-EU-Q16 score >8 and ≤12, HLS-EU-Q6 score >2 and ≤3), and adequate (HLS-EU-Q16 score >12, HLS-EU-Q6 score >3) [18,20,53]. The association between the overall HLS-EU-Q16 and HLS-EU-Q6 scores was estimated with the Spearman correlation coefficient, and the kappa coefficient was used to evaluate agreement (acceptable if kappa>0.6, excellent if >0.8) between HL levels determined by the HLS-EU-Q16 and HLS-EU-Q6 [54]. Spearman coefficients were also used to evaluate the association of both HLS-EU overall scores with the FCCHL functional, communicative and critical HL scores, and the kappa coefficient was used to evaluate the agreement between the HL levels obtained for each patient by the HLS-EU-Q16 and HLS-EU-Q6 with the level evaluated by the physician.

To determine the questionnaires’ convergent and discriminant validity, comparisons were made between patients depending on their education level, perceived health status, and financial situation, and HL as evaluated by their physician. Lower HL was expected for less educated patients, those with poorer perceived health status, poorer perceived financial situation, and low physician-assessed HL level [55–58]. These *a priori* hypotheses were tested with Mann-Whitney tests. The kappa coefficient was also used to evaluate the agreement between the HL level determined with the HLS-EU-Q16 and HLS-EU-Q6, and the physician-assessed HL level. Analyses were performed with the Stata v.14 (data management and basic statistics), RUMM2030 (Rasch analyses), and Mplus v7.4 (CFA) software [59–61].

## Results

### Subjects

Of the 372 patients who were approached for the study, 343 agreed to participate (response rate: 92%); 26 (8%) were subsequently excluded due to a missing answer on one or more of the HLS-EU-Q16 items. Table 1 summarizes the socio-demographic characteristics of the remaining 317 patients; 207 (65%) were women, their mean age was 53 (±18) years and 188 (59%) had a post-secondary education level. In all, 216 (68%) assessed their financial situation as “very comfortable” or “relatively comfortable” and 208 (66%) rated their health as "good", "very good" or "excellent".

**Table 1:** Characteristics of the sample (N = 317).

Characteristic	N (%)
Age	
≤ 40 years	88 (28)
41 to 60 years	106 (34)
> 60 years	115 (37)
Sex (Male)	110 (35)
Lives with a partner	182 (58)
At least a child < 18 years old at home	102 (32)
French as native language	256 (82)
Education	
Primary or none	73 (24)
Secondary	55 (17)
Post-secondary	188 (59)
Profession	
Shopkeepers and crafts workers	13 (4)
Professionals and managers	132 (42)
Office, sales, and service employees	66 (21)
Skilled or unskilled manual workers	15 (5)
Intermediate white-collar workers	70 (23)
Never worked	15 (5)

### Psychometric properties of the French version of the HLS-EU-Q16 and HL-SEU-Q6

#### Descriptive analyses and floor and ceiling effects

None of the HLS-EU-Q16 items had floor or ceiling effects when the 4-point Likert scale was used (S2 Table). After they were dichotomized, however, ceiling effects were observed for four items: item 3 (understanding your doctor), 4 (understanding your doctor’s or pharmacist’s instructions), 7 (following your doctor’s or pharmacist’s instructions), and 10 (understanding why you need health screening tests). The distributions of the scores on the HLS-EU-Q16 and HLS-EU-Q6 are reported in Table 2. A ceiling effect was observed for the HLS-EU-Q16 score, with 80 (25%) patients scoring 16. When the scores were categorised, the HL level was defined as inadequate for 26 (8%) and 16 (5%) subjects, problematic for 106 (33%) and 218 (69%), and adequate for 185 (58%) and 83 (26%) with the HLS-EU-Q16 and HLS-EU-Q6, respectively.

**Table 2:** Distribution of the scores on European Health Literacy Survey Questionnaire with 16 items (HLS-EU-Q16) and with 6 items (HLS-EU-Q6) in the overall sample and according to physician’s evaluation of patient health literacy (HL), education level, perceived health status, and perceived financial situation.

	N	HLS-EU-Q16			HLS-EU-Q6		
		Mean (SD)	Median (Q1 –Q3)	p-value	Mean (SD)	Median (Q1 –Q3)	p-value
Total	317	12.8 (3.0)	13 (11–16)		2.8 (0.5)	2.8 (2.2–3.2)	
Patient HL evaluated by the physician							
Inadequate	26	10.3 (4.5)	11 (8–14)	0.002	2.6 (0.6)	2.7 (2.3–3)	0.033
Medium	81	12.5 (2.9)	13 (10–15)		2.8 (0.5)	2.8 (2.3–3)	
Satisfactory	179	13.2 (2.8)	14 (11–16)		2.9 (0.5)	2.8 (2.5–3.2)	
Education							
Primary	73	12.3 (3.6)	13 (11–15)	0.480	2.8 (0.6)	2.8 (2.5–3)	0.753
Secondary	55	13.2 (2.5)	14 (12–15)		2.9 (0.5)	2.8 (2.7–3.2)	
Post-secondary	188	12.8 (2.9)	13 (11–16)		2.8 (0.5)	2.8 (2.5–3.1)	
Perceived health status							
Excellent, very good	78	13.4 (2.6)	14 (12–16)	0.073	2.9 (0.5)	2.8 (2.5–3.2)	0.539
Good	130	12.9 (2.5)	13 (11–15)		2.8 (0.5)	2.7 (2.5–3)	
Not bad, mediocre	107	12.1 (3.7)	13 (9–15)		2.8 (0.6)	2.8 (2.3–3.2)	
Perceived financial situation							
Very comfortable	32	13.7 (2.7)	15 (11.5–16)	0.031	3.2 (0.6)	3 (2.7–3.8)	0.017
Relatively comfortable	184	13.0 (2.9)	13 (11–16)		2.8 (0.5)	2.8 (2.5–3.1)	
Just about managing	85	12.3 (3.2)	13 (11–15)		2.7 (0.5)	2.7 (2.3–3)	
Not really managing/not managing	15	11.1 (4.0)	11 (8–15)		2.8 (0.8)	2.8 (2.2–3.2)	

#### Rasch analyses and study of the measurement invariance of the HLS-EU-Q16

Loevinger’s H coefficients confirmed the unidimensionality, local independence and monotonicity hypotheses, except for the H coefficient (0.28) associated with item 1 (finding information on treatments). The overall Chi^2^ test *P*-value for the Rasch model fit was 0.08. Standardized fit residuals and Chi^2^ tests indicated that the Rasch model had a good fit at the item level, as summarized in Table 3. Item difficulty varied from -2.42 to 2.18, and the latent trait (HL) level of more than 40% of the sample was higher than the highest item’s difficulty, as shown on the person-item map (S1 Fig).

**Table 3:** Item fit of the French short version of the European Health Literacy Survey Questionnaire with 16 items (HLS-EU-Q16), with the Rasch model.

Item	Fit into the Rasch model			
On a scale from very easy to very difficult, how easy would you say it is to:	Difficulty	Fit residual^a^	Chi square	P-value^b^
1—find information on treatments of illnesses that concern you?	0.05	0.92	6.29	0.179
2—find out where to get professional help when you are ill?	-0.49	-0.16	3.53	0.473
3—understand what your doctor says to you?	-1.88	0.08	5.35	0.253
4—understand your doctor’s or pharmacist’s instruction on how to take a prescribed medicine?	-2.42	-1.00	1.82	0.769
5—judge when you may need to get a second opinion from another doctor?	0.88	0.74	3.59	0.464
6—use information the doctor gives you to make decisions about your illness?	-0.63	-0.90	4.99	0.288
7—follow instructions from your doctor or pharmacist?	-1.70	-1.32	4.11	0.392
8—find information on how to manage mental health problems like stress or depression?	1.46	0.64	3.70	0.448
9—understand health warnings about behavior such as smoking, low physical activity and drinking too much?	-0.86	0.59	6.19	0.185
10—understand why you need health screenings?	-1.56	0.09	2.04	0.729
11—judge if the information on health risks in the media is reliable?	2.18	-0.47	4.21	0.378
12—decide how you can protect yourself from illness based on information in the media?	1.81	-1.47	15.09	0.005
13—find out about activities that are good for your mental well-being?	0.93	0.43	3.54	0.472
14—understand advice on health from family members or friends?	0.52	0.64	8.53	0.074
15—understand information in the media on how to get healthier?	1.42	-1.04	5.55	0.236
16—judge which everyday behavior is related to your health?	0.29	0.01	2.28	0.685

^a^Fit residual values between ±2.5 indicate a good fit to the Rasch model

^b^Bonferroni adjustment: significant if *P*-value<0.0031

Table 4 presents the results from the DIF analyses. Item 1 (finding information on treatments) showed meaningful DIF across sex can be interpreted as follow: if men and women have the same HL level, men respond more often than women that it is difficult to find information on treatments of illnesses that concern them. Three items (item 3: understanding what your doctor says; item 5: judging when you may need to get a second opinion; and item 14: understand advice on health from family members or friends) showed meaningful DIF across age. At the same HL level, older people respond more often than younger that it is difficult to understand what the doctor says and to judge when they need to get a second opinion. Persons between 41 and 60 respond more often that it is easy to understand advice on health from relatives and friends compared to younger or older people. Education level was associated with a meaningful DIF: two items were easier for more educated patients (item 1: finding information on treatments, and item 6: using information the doctor gives you to make decisions), and two more difficult (item 11: judging if the information on health risks in the media is reliable, and item 12: deciding how you can protect yourself from illness based on information in the media). At the same HL level, more educated subjects answer more often that it is difficult to find information on treatments and to use information given by the doctor to make decisions. To the contrary, they answer less that it is difficult to judge the reliability of the information in the media and to decide how to protect themselves based on information in the media. As DIF was not in the same direction for these five items, a higher HLS-EU-Q16 score was expected for more educated than for less educated subjects when the latent trait (HL level) was low, while a lower score was expected when the HL level was high (S2 Fig). The expected difference of the HLS-EU-Q16 score due to DIF across education levels reached a maximum of 0.54 points between the primary and post-secondary education levels for a latent trait level of 2 logit (i.e., an expected score of 13.55 and of 13.01 in the primary and post-secondary education levels respectively).

**Table 4:** Items from the HLS-EU-Q16 with meaningful differential item functioning across sex, age, or education level.

HLSEU Item		Differential Item functioning		
	p-value	Item’s difficulty in group categories		
**SEX**		Male	Female	
1—find information on treatments of illnesses that concern you?	0.042	-0.58	0.30	
**AGE**		18 to 40 years	41 to 60 years	61 years and older
3—understand what your doctor says to you?	0.035	-0.74	-2.90	-2.32
5—judge when you may need to get a second opinion from another doctor?	0.002	1.69	0.74	0.47
14—understand advice on health from family members or friends?	0.015	0.01	1.10	0.50
**EDUCATION**		Primary	Secondary	Post-secondary
1—find information on treatments of illnesses that concern you?	0.019	0.15	0.30	-0.57
6—use information the doctor gives you to make decisions about your illness?	0.017	-0.61	-0.16	-1.35
11—judge if the information on health risks in the media is reliable?	0.016	1.13	1.97	2.27
12—decide how you can protect yourself from illness based on information in the media?	<0.001	0.72	0.93	2.14
16—judge which everyday behaviour is related to your health?	0.043^a^	-0.20	-0.48	0.29

^a^Non-uniform differential item functioning

#### CFA of the HL-SEU-Q6 questionnaire

Indices of fit for the one-factor CFA model applied on the reversed 4-point Likert scale were: CFI = 0.948; TLI = 0.913, and RMSEA = 0.176 (90% confidence interval, 0.145; 0.208). Computational issues (non-positive information matrix) precluded a reliable assessment of fit indices for the two-order CFA model.

#### Internal consistency

The Cronbach alpha coefficients were 0.81 and 0.83 for the HLS-EU-Q16 and the HLS-EU-Q6, respectively.

#### Concurrent validity

The Spearman correlation between the HLS-EU-Q16 and HLS-EU-Q6 scores was 0.88. In contrast, the agreement between HL levels defined by both of these versions was poor, with a Kappa coefficient equal to 0.36. In addition, the Spearman correlation coefficients of the scores on both these versions with the functional, communicative and critical HL scales of the FCCHL were statistically significant (*P*-value<0.05) but all below 0.3 (range: 0.11–0.29).

#### Convergent and discriminant validity

Results from the *a priori* hypotheses-tests are shown in Table 2. No significant differences were observed between patients according to their education level or perceived health status. A trend was observed by which the HLS-EU-Q16 score decreased with perceived health status, but not for the HLS-EU-Q6. HL measures were not associated with education level, even when the score was computed without the five items affected by DIF concerning education. On the other hand, a significant association was found between HL and perceived financial situation. Physicians evaluated the HL as insufficient for 26 (9%) patients, medium for 81 (28%) and satisfactory for 179 (63%). On average, the overall HLS-EU-Q16 and HLS-EU-Q6 scores were higher with higher physician-assessed HL (*P*-values = 0.002 and 0.033, respectively) (Table 4). Nonetheless, the agreement between the HL categories as evaluated by physicians and using the questionnaires was poor, with Kappa coefficients equal to 0.10 for the HLS-EU-Q16 and 0.06 for the HLS-EU-Q6.

## Discussion

Transcultural adaptation and validation of the short forms of the HLS-EU questionnaire are necessary steps before they can be used to measure HL at the population level among French-speaking subjects and then to compare HL levels across populations, which was the primary aim of the HLS-EU study [17].

Our results indicate that the French version of the HLS-EU-Q16 is Rasch homogenous, which is a highly recommended property for composite measurement scales. Its internal consistency is also satisfactory. On the other hand, our analyses also revealed certain limitations that suggest the need for some caution when using this form of the questionnaire. First, four items showed ceiling effects when dichotomized, which suggests that these items are not sufficiently discriminant in this population. The Rasch analysis and the ceiling effect observed for the total score are consistent with this observation and indicate that the scale based on dichotomization of the items is not sufficiently discriminatory for use among subjects with high levels of HL. As such, the French version of the questionnaire appears more appropriate for the study of people and groups with relatively low levels of HL, to discriminate between them. This finding also raises the question whether any HLSEU-Q16 items should be treated as binary items, since when scored on a four-point Likert scale, they did not show any ceiling effects.

Second, measurement invariance did not hold as DIF was observed for sex, age, and education level. Different explanations can enlighten this phenomenon. Women are more concerned about their health than men. They have more medical encounters and are more likely to seek medical treatment. Moreover, physicians spend more time with female patients and give them more explanations [62]. This specific relationship to the health care system may explain why women declare that finding information on treatments is easier than men.

Two items were more difficult for older people (understand what the doctor says and to judge when they need to get a second opinion). This may be due, for example, to age related hearing or cognitive impairments that make understanding and judgement more complicated without modifying the HL level at all. The finding that more educated subjects answer more often that it is difficult to find information on treatments and to use information given by the doctor to make decisions may be related to the known relationship between education and information seeking or preference for decision making [63,64]. While some degree of DIF is often found in questionnaire validation studies, ignoring the presence of this phenomenon could lead to biased results [51]. The DIF is particularly noticeable for education level, where it affects five items in opposite directions. Because the score difference across education levels due to DIF amounts to 0.5 units on the HLS-EU-Q16 total score, it can lead to underestimating the educational gradient in HL. The overall good fit of the Rasch model means that it is the same measured latent trait (HL) across groups but the presence of DIF signifies that it is not measured in the same way across groups. To counteract this bias, it is recommended that studies using the questionnaire in populations that vary in terms of sex, age, or (especially) education level perform sensitivity analyses by calculating the HLS-EU-Q16 scores with and without the items showing DIF. To our knowledge, this study is the first to investigate DIF for the HLS-EU questionnaire. Evidence of the amplitude of the biases this phenomenon may produce is necessary to allow a well-informed use of the questionnaire. We therefore recommend that it be investigated in other language versions.

The results for convergent and divergent validity were consistent with our a priori hypotheses, except for that concerning education level, which was not related to the total score on the HLS-EU-Q16. This may be due to the readability level of the French version of this questionnaire, which corresponded to that of a university undergraduate. It might have induced a selection bias by discouraging respondents with lower education levels from completing the questionnaire.

Finally, correlations between the overall HLS-EU-Q16 score and the FCCHL scores were very low, as was the agreement between the HL categories determined by the HLS-EU-Q16 and by the physician. This suggests that what is measured by the HLS-EU-Q16 is different from HL as measured by the FCCHL or as conceptualized by French physicians. With regard to the latter, nonetheless, we note that many of the doctors who participated in the study were unfamiliar with the concept of HL; this lack of knowledge probably explains the low level of agreement. Correlation with the FCCHL was also low, perhaps because the latter questionnaire focuses mainly on HL in the patient-clinician interaction, and less on the health information processing skills of accessing, understanding, appraising, and applying health information in disease prevention and health promotion, which are important objects of the HLS-EU-Q. Further research exploring the links and differences between the constructs measured by the various existing health literacy measurement instruments would be helpful in choosing the most suitable questionnaire for each study.

The validity of the French version of the HLS-EU-Q6 could not be established, due to the poor fit of the one-factor CFA model and our inability to estimate the fit of a two-order CFA model reliably, due to computation issues. The Spearman correlation between the overall HLS-EU-Q16 and HLS-EU-Q6 scores was nevertheless high, suggesting that both tools measure the same construct. On the other hand, the agreement between HL levels measured by the HLS-EU-Q16 and HLS-EU-Q6 was poor, which suggests that different thresholds may be needed to categorize the overall HLS-EU-Q6 score. Moreover, the results from the analyses regarding convergent and discriminant validity were not convincing. To our knowledge, no studies have examined the psychometric properties of this short version, in any language, although it has already been used in some epidemiological studies [21,22]. Further studies should be planned to evaluate the validity of the HLS-EU-Q6 in other languages.

This study nonetheless has limitations. The sample size could be perceived as a weakness, although a sample size of 200 persons has been recommended for Rasch analysis [65]. In addition, although the entire French population has access to primary care without social differences, the method for participant recruitment via the waiting room probably resulted in over representing women and elderly people in the sample. Accordingly, inadequate specification of the psychometric properties of the French version of the HLS-EU short versions due to this selection bias cannot be ruled out. Moreover, we used a sample of participants from the Paris area, so our results must be replicated on samples of subjects from other French-speaking areas to evaluate their robustness. Finally, the use of self-administered questionnaires is limited to people who can read French well, and ad hoc studies should be planned to develop self-administered measurement tools that can be used in populations with poor reading skills.

## Conclusion

Despite these limitations, we conclude that the psychometric properties of the French version of the HLS-EU-Q16 enable its use in surveys of health literacy, provided that the population surveyed has sufficient reading skills (preferably not lower than high-school level), and that it is mainly suitable to discriminate between subjects with low to average HL level. Sensitivity analyses should also be performed to evaluate the role of potential measurement bias due to DIF related to sex, age, and education level in the HLS-EU-Q16. Furthermore, as measurement invariance has rarely been studied in the field of HL assessment [13], we suggest that further studies should assess this property in every language version of the HLS-EU questionnaires, as well as for other HL measurement instruments commonly used, such as the HLQ, REALM and TOFHLA. Finally, the validity of the HLS-EU-Q6 could not be established in this study.

## Supporting information

S1 Table Items from the European Health Literacy Survey Questionnaire short forms, 16 items (HLS-EU-Q16) and 6 items (HLS-EU-Q6, in bold, and the health contexts and health-information-processing skills to which they apply. (DOCX)

S2 Table Frequencies (%) of responses to the 16 items of the European Health Literacy Survey Questionnaire short forms, HLS-EU-Q16 and HLS-EU-Q6 (in bold), in the sample (N = 317). (DOCX)

S3 Table HLSEUvalidFrench.xls is the database used in this study. (XLS)

S1 Fig Rasch person-item map of the European Health Literacy Survey Questionnaire with 16 items (HLS-EU-Q16) in the sample (N = 317). A higher (positive) location value indicates higher health literacy of the persons or greater item difficulty (logit scale).
(DOCX)

S2 Fig Expected score on the European Health Literacy Survey Questionnaire with 16 items depending on education level and expected difference in score due to differential item functioning across education levels (reference “Post-secondary”) as a function of latent trait (as an example, for subjects with a health literacy level at 2 logits on the latent trait scale, their scores are expected to be 13.6, 13.3 and 13.0 on average, respectively, in the primary, secondary and post-secondary education level groups). (DOCX)

S1 Text French version of the European Health Literacy Survey Questionnaire short form with 16 items (HLS-EU-Q16). (DOCX)
