# Supplementary material for: Validity and measurement invariance across sex, age, and education level of the French short versions of the European Health Literacy Survey Questionnaire
Source: PLoS One. 2018 Dec 6;13(12):e0208091. doi: 10.1371/journal.pone.0208091 (PMC6283623; doi:10.1371/journal.pone.0208091)
Supplement: S1 Table — (DOCX) [file pone.0208091.s001.docx]

**S1 Table**. Items from the European Health Literacy Survey Questionnaire short forms, 16 items (HLSEU16) and 6 items (HLSEU6, in bold), and health contexts and health-information-processing competencies to which they apply.

| **Items** |  | **Health contexts** | | |  | **Health-information-processing competencies** | | | |
| --- | --- | --- | --- | --- | --- | --- | --- | --- | --- |
| **On a scale from very easy to very difficult, how easy would you say it is to: …** |  | **Healthcare** | **Disease prevention** | **Health promotion** |  | **Accessing** | **Understanding** | **Appraising** | **Applying** |
| 1 - find information on treatments of illnesses that concern you? |  | X |  |  |  | X |  |  |  |
| 2 - find out where to get professional help when you are ill? |  | X |  |  |  | X |  |  |  |
| 3 - understand what your doctor says to you? |  | X |  |  |  |  | X |  |  |
| 4 - understand your doctor’s or pharmacist’s instruction on how to take a prescribed medicine? |  | X |  |  |  |  | X |  |  |
| **5 - judge when you may need to get a second opinion from another doctor?** |  | **X** |  |  |  |  |  | **X** |  |
| **6 - use information the doctor gives you to make decisions about your illness?** |  | **X** |  |  |  |  |  |  | **X** |
| 7 - follow instructions from your doctor or pharmacist? |  | x |  |  |  |  |  |  | X |
| **8 - find information on how to manage mental health problems like stress or depression?** |  |  | **X** |  |  | **X** |  |  |  |
| 9 - understand health warnings about behavior such as smoking, low physical activity and drinking too much? |  |  | X |  |  |  | X |  |  |
| 10 - understand why you need health screenings? |  |  | X |  |  |  | X |  |  |
| **11 - judge if the information on health risks in the media is reliable?** |  |  | **X** |  |  |  |  | **X** |  |

**S1 Table**. Continued.

| **Items** |  | **Health contexts** | | |  | **Health-information-processing competencies** | | | |
| --- | --- | --- | --- | --- | --- | --- | --- | --- | --- |
| **On a scale from very easy to very difficult, how easy would you say it is to: …** |  | **Healthcare** | **Disease prevention** | **Health promotion** |  | **Accessing** | **Understanding** | **Appraising** | **Applying** |
| 12 - decide how you can protect yourself from illness based on information in the media? |  |  | X |  |  |  |  |  | X |
| **13 - find out about activities that are good for your mental well-being?** |  |  |  | **X** |  | **x** |  |  |  |
| 14 - understand advice on health from family members or friends? |  |  |  | X |  |  | X |  |  |
| **15 - understand information in the media on how to get healthier?** |  |  |  | **X** |  |  | **X** |  |  |
| 16 - judge which everyday behavior is related to your health? |  |  |  | x |  |  |  | X |  |
