# Supplementary material for: Validity and measurement invariance across sex, age, and education level of the French short versions of the European Health Literacy Survey Questionnaire
Source: PLoS One. 2018 Dec 6;13(12):e0208091. doi: 10.1371/journal.pone.0208091 (PMC6283623; doi:10.1371/journal.pone.0208091)
Supplement: S2 Table — (DOCX) [file pone.0208091.s002.docx]

**S2 Table**. Frequencies (%) of responses to the 16 items of the European Health Literacy Survey Questionnaire short forms, HLSEU16 and HLSEU6 (in bold), in the sample (N=317)

| Item | Very easy | Fairly easy | Fairly difficult | Very difficult |
| --- | --- | --- | --- | --- |
| 1 - find information on treatments of illnesses that concern you? | 63 (20) | 204 (64) | 44 (14) | 6 (2) |
| 2 - find out where to get professional help when you are ill? | 76 (24) | 207 (65) | 32 (10) | 2 (1) |
| 3 - understand what your doctor says to you? | 129 (41) | 176 (56) | 11 (3) | 1 (0) |
| 4 - understand your doctor’s or pharmacist’s instruction on how to take a prescribed medicine? | 160 (51) | 148 (47) | 7 (2) | 2 (1) |
| **5 - judge when you may need to get a second opinion from another doctor?** | 65 (21) | 166 (52) | 78 (25) | 8 (3) |
| **6 - use information the doctor gives you to make decisions about your illness?** | 90 (28) | 196 (62) | 26 (8) | 5 (2) |
| 7 - follow instructions from your doctor or pharmacist? | 140 (44) | 162 (51) | 12 (4) | 3 (1) |
| **8 - find information on how to manage mental health problems like stress or depression?** | 50 (16) | 154 (49) | 101 (32) | 12 (4) |
| 9 - understand health warnings about behavior such as smoking, low physical activity and drinking too much? | 146 (46) | 144 (45) | 21 (7) | 6 (2) |
| 10 - understand why you need health screenings? | 139 (44) | 163 (51) | 13 (4) | 2 (1) |
| **11 - judge if the information on health risks in the media is reliable?** | 47 (15) | 116 (37) | 130 (41) | 24 (8) |
| 12 - decide how you can protect yourself from illness based on information in the media? | 44 (14) | 138 (44) | 119 (38) | 16 (5) |
| **13 - find out about activities that are good for your mental well-being?** | 52 (16) | 176 (56) | 83 (26) | 6 (2) |
| 14 - understand advice on health from family members or friends? | 64 (20) | 181 (57) | 60 (19) | 12 (4) |
| **15 - understand information in the media on how to get healthier?** | 52 (16) | 148 (47) | 105 (33) | 12 (4) |
| 16 - judge which everyday behavior is related to your health? | 69 (22) | 185 (58) | 57 (18) | 6 (2) |
