# Supplementary material for: Validity and measurement invariance across sex, age, and education level of the French short versions of the European Health Literacy Survey Questionnaire
Source: PLoS One. 2018 Dec 6;13(12):e0208091. doi: 10.1371/journal.pone.0208091 (PMC6283623; doi:10.1371/journal.pone.0208091)
Supplement: S1 Fig — A higher (positive) location value indicates higher health literacy of the persons or greater item difficulty (logit scale). (DOCX) [file pone.0208091.s004.docx]

**S1 Fig.** Rasch person-item map of the European Health Literacy Survey Questionnaire with 16 items (HLSEU16) in the sample (N=317). A higher (positive) location value indicates higher health literacy of the persons or greater difficulty of the item (logit scale).

**
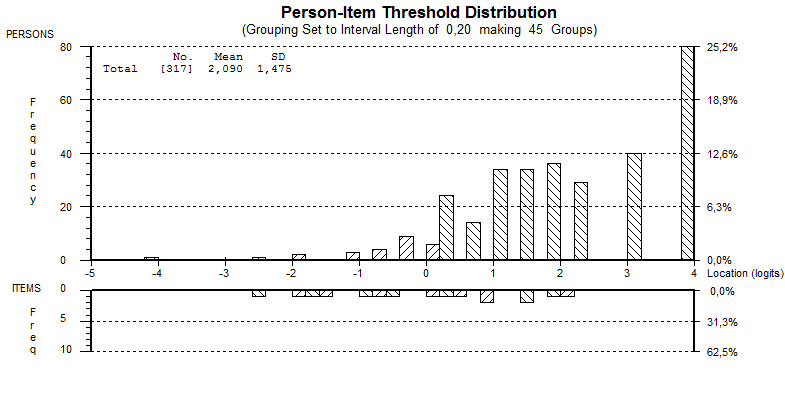
**
