# Supplementary material for: Validity and measurement invariance across sex, age, and education level of the French short versions of the European Health Literacy Survey Questionnaire
Source: PLoS One. 2018 Dec 6;13(12):e0208091. doi: 10.1371/journal.pone.0208091 (PMC6283623; doi:10.1371/journal.pone.0208091)
Supplement: S2 Fig — (DOCX) [file pone.0208091.s005.docx]

**S2 Fig.** Expected score to the European Health Literacy Survey Questionnaire with 16 items depending on education level and expected difference in score due to differential item functioning across education levels (reference “Post-secondary”) as a function of latent trait (as an example, for subjects with a health literacy level at 2 logits on the latent trait scale, their score is expected to be 13.6, 13.3 and 13.0 on average in the primary, secondary and post-secondary education level group respectively).

**
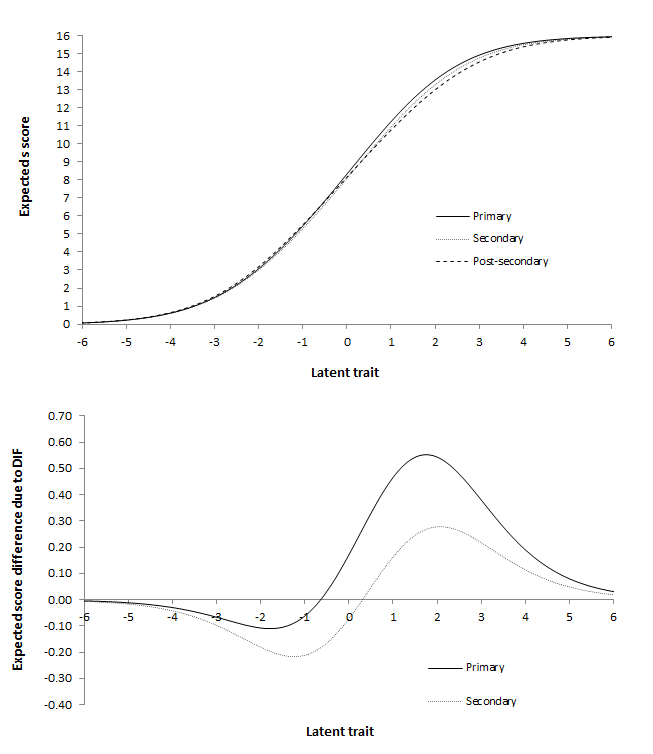
**
