# Supplementary material for: Validity and measurement invariance across sex, age, and education level of the French short versions of the European Health Literacy Survey Questionnaire
Source: PLoS One. 2018 Dec 6;13(12):e0208091. doi: 10.1371/journal.pone.0208091 (PMC6283623; doi:10.1371/journal.pone.0208091)
Supplement: S1 Text — (DOCX) [file pone.0208091.s006.docx]

**S1 Text**. French version of the European Health Literacy Survey Questionnaire short form with 16 items (HLSEU16)

**Indiquez, sur une échelle de très facile à très difficile, dans quelle mesure il est facile pour vous de...**

|  | très facile | facile | difficile | très difficile |
| --- | --- | --- | --- | --- |
| ... trouver des informations sur les traitements des maladies qui vous concernent ? | ❑ | ❑ | ❑ | ❑ |
| … savoir où obtenir l’aide d’un professionnel quand vous êtes malade ? | ❑ | ❑ | ❑ | ❑ |
| … comprendre ce que vous dit votre médecin ? | ❑ | ❑ | ❑ | ❑ |
| … comprendre les consignes de votre médecin ou de votre pharmacien sur la manière de prendre vos médicaments ? | ❑ | ❑ | ❑ | ❑ |
| … savoir quand il serait utile d’avoir l’avis d’un autre médecin ? | ❑ | ❑ | ❑ | ❑ |
| … utiliser les informations que le médecin vous donne pour prendre des décisions concernant votre maladie ? | ❑ | ❑ | ❑ | ❑ |
| … suivre les consignes de votre médecin ou de votre pharmacien ? | ❑ | ❑ | ❑ | ❑ |
| … trouver des informations sur comment faire en cas de problèmes psychologiques comme le stress ou la dépression ? | ❑ | ❑ | ❑ | ❑ |
| … comprendre les mises en gardes concernant l’impact sur la santé de certains comportements comme fumer, ne pas faire assez d’exercices et boire trop ? | ❑ | ❑ | ❑ | ❑ |

**Tournez la page s’il vous plaît…**

**Indiquez, sur une échelle de très facile à très difficile, dans quelle mesure il est facile pour vous de...**

|  | très facile | facile | difficile | très difficile |
| --- | --- | --- | --- | --- |
| … comprendre l’intérêt des dépistages ? | ❑ | ❑ | ❑ | ❑ |
| … évaluer la fiabilité des informations disponibles dans les médias sur ce qui est dangereux pour la santé ? | ❑ | ❑ | ❑ | ❑ |
| … savoir comment vous préserver des maladies à partir des informations disponibles dans les médias ? | ❑ | ❑ | ❑ | ❑ |
| … vous renseigner sur les activités bénéfiques pour votre bien être psychologique ? | ❑ | ❑ | ❑ | ❑ |
| … comprendre les conseils de votre famille ou de vos amis en matière de santé ? | ❑ | ❑ | ❑ | ❑ |
| … comprendre les informations disponibles dans les médias pour être en meilleure santé ? | ❑ | ❑ | ❑ | ❑ |
| … identifier quels sont les comportements de votre vie de tous les jours qui ont un impact sur votre santé ? | ❑ | ❑ | ❑ | ❑ |

**Fin du questionnaire.**
